# Impact of Selection and Demography on the Diffusion of Lactase Persistence

**DOI:** 10.1371/journal.pone.0006369

**Published:** 2009-07-24

**Authors:** Pascale Gerbault, Céline Moret, Mathias Currat, Alicia Sanchez-Mazas

**Affiliations:** Laboratory of Anthropology, Genetics and Peopling History (AGP), Department of Anthropology and Ecology, University of Geneva, Geneva, Switzerland; University of Utah, United States of America

## Abstract

**Background:**

The lactase enzyme allows lactose digestion in fresh milk. Its activity strongly decreases after the weaning phase in most humans, but persists at a high frequency in Europe and some nomadic populations. Two hypotheses are usually proposed to explain the particular distribution of the lactase persistence phenotype. The *gene-culture coevolution* hypothesis supposes a nutritional advantage of lactose digestion in pastoral populations. The *calcium assimilation* hypothesis suggests that carriers of the lactase persistence allele(s) (LCT*P) are favoured in high-latitude regions, where sunshine is insufficient to allow accurate vitamin-D synthesis. In this work, we test the validity of these two hypotheses on a large worldwide dataset of lactase persistence frequencies by using several complementary approaches.

**Methodology:**

We first analyse the distribution of lactase persistence in various continents in relation to geographic variation, pastoralism levels, and the genetic patterns observed for other independent polymorphisms. Then we use computer simulations and a large database of archaeological dates for the introduction of domestication to explore the evolution of these frequencies in Europe according to different demographic scenarios and selection intensities.

**Conclusions:**

Our results show that *gene-culture coevolution* is a likely hypothesis in Africa as high LCT*P frequencies are preferentially found in pastoral populations. In Europe, we show that population history played an important role in the diffusion of lactase persistence over the continent. Moreover, selection pressure on lactase persistence has been very high in the North-western part of the continent, by contrast to the South-eastern part where genetic drift alone can explain the observed frequencies. This selection pressure increasing with latitude is highly compatible with the *calcium assimilation* hypothesis while the *gene-culture coevolution* hypothesis cannot be ruled out if a positively selected lactase gene was carried at the front of the expansion wave during the Neolithic transition in Europe.

## Introduction

Lactase is an enzyme that allows lactose digestion in fresh milk. Its activity strongly decreases after the weaning phase in most humans. However, in many European individuals and in people from various populations of other continents, lactase is still active throughout adult life [1]. This dominantly inherited genetic trait is called *lactase persistence* and at least three mutations are tightly associated with it: *−13,910 C/T* (generally linked to *−22,018 G/A*) in northern Europe (100% association) [2], [3], *−14,010 G/C* in East Africa and *−13,915 T/G* in the Middle-East/North Africa [4], [5]. In this study, we will use *LP* to refer to the *lactase persistence* phenotype, *LCT* to refer to the *lactase gene* and LCT*P to refer to the *lactase persistence* associated allele(s).

The particular distribution of lactase persistence throughout the world indicates that this trait evolved under strong positive selection [5], [6], [7]. Two main hypotheses have been proposed: *gene-culture coevolution* (*gcc*) [8], [9], which suggests that lactose digestion confers a nutritional advantage to milk-consuming (e.g. pastoralist) populations; and *calcium assimilation* (*cal*) [10], which proposes that carriers of LCT*P are favoured in high-latitude regions, where lactose would substitute vitamin-D (deficient when sunlight is low) to allow accurate calcium assimilation, thus preventing rickets. Holden and Mace [11] studied the two mentioned hypotheses and a third one, proposing that LP was favoured in highly arid environments, where people would have drunk milk to prevent dehydration [12], [13]. Their conclusion was that LP is an adaptation to pastoralism, in agreement with the *gcc* model, while no evidence for the other hypotheses was found. Compatible with this theory, Coelho et al. [14] suggested that −13,910*T originated in Eurasia before the Neolithic Era, and observed a significant departure from neutrality of this variant in the few populations examined. On the other hand, these studies do not explain the significant correlation found between LP frequencies and latitude in Europe [15], as predicted by the *cal* model.

The aim of the present work was to test the relevance of the two main hypotheses associated with lactase persistence, *gcc* and *cal*, using a large database of LP frequencies (115 populations worldwide, Table S1 in Supplementary Information). In a first step, we analysed the correlation between LCT*P variation, geography, and the variation of other independent polymorphisms at different geographic scales, to check whether LCT*P genetic patterns were really peculiar or could have been shaped, at least in part, by population history and demography. We also analysed the correlation between LCT*P frequencies and pastoralism levels in Africa. In a second step, we used computer simulation to investigate a possible effect of positive selection in Europe, where LP variation is particularly sharp. Our algorithm considers various parameters (genetic drift, demographic growth, positive selection, and the time elapsed since the likely beginning of milk consumption), and takes into account two alternative models of Neolithic diffusion: demic and cultural [16]. According to our results, the *gcc* hypothesis fits better the observed data in Africa while the *cal* hypothesis is compatible with observed data in Europe.

## Results

### Correlation between genetics, geography and pastoralism levels

We first tested the correlation between genetic and geographic variation for LCT*P, both at the global and at continental scales (Table 1). Our goal was to assess whether LCT*P could have evolved neutrally. Indeed, a significant and positive correlation is expected between genetic and geographic variation under a simple model of isolation by distance [17], [18]. Of course, as such a correlation may also be observed under some non-neutral models (like selection due to environmental factors showing geographic clines, or geographic spread of a beneficial allele arising in a single location and spreading outwards), additional analyses would be required to confirm neutrality. On the other hand, no correlation at all between genetic and geographic variation would indicate that migration history did not leave any signature on *LCT* genetic patterns.

**Table 1:** Correlation coefficients between genetics and geography.

Region	n	r_gen,geo_	P val.	r_LCT*P-lat_	P val.	r_LCT*P-long_	P val.
World Scale	115	0.050	0.0400	0.274	<0.0001	−0.080	0.003
Europe	33	0.260	0.0001	0.400	0.0005	−0.010	0.568
Europe (indo-europeans only)	26	0.420	0.0000	0.840	<0.0001	−0.310	0.006
Africa	38	0.016	0.1700	0.329	0.0010	0.134	0.048
Near East &East Asia	34	0.016	0.3600	−0.041	0.2560	−0.046	0.230

*n*: number of populations; *r*_gen,geo_: correlation coefficient between genetic and geographic distances; *r*_LCT*P-lat_: correlation coefficient between LCT*P frequency and latitude; r_LCT*P-long_:correlation coefficient between LCT*P frequency and longitude; P.val.: P-value for significance. Population used are listed in Table S1.

### Worldwide level

When all 115 worldwide populations were considered, a weak but significant (at level 5%) correlation was found between genetic and geographic distances (*r* = 0.05, *p* = 0.04, Table 1). The correlation coefficient between LCT*P frequencies and latitude was highly significant (*r* = 0.274, *p*<0.0001). However, this result was merely due to a sharp contrast existing between LCT*P frequencies observed in Europe (high to very high) and those observed in sub-Saharan Africa (low to very low), rather than to a south to north continuum of frequencies supporting an isolation by distance model (results of spatial autocorrelation analysis not shown). The correlation between LCT*P frequencies and longitude was very low but negative and significant (*r* = −0.08, *p* = 0.003), which was reflecting the higher frequencies observed in Europe and Africa, compared to East Asia. Globally, while these results were not supporting any peculiar model of natural selection, they were indicating that the evolution of LCT*P was probably not purely neutral.

### Continental levels

When looking at continental scales, we did not observe any significant correlation between genetic and geographic variation in Asia (i.e. Near East & East Asia, Table 1). This was explained by a heterogeneous distribution of LCT*P in this continent (from 0 to 0.626 in the Near East and India, and very low in East Asia). The gene did probably not evolve neutrally there, but no clear pattern appeared as to allow further explanations. Moreover, we could not have tested the *gcc* hypothesis as we did for Africa (see below) due to a lack of data on pastoralism levels in Asia. Note also that for Oceania and the Americas, the number of available samples was too low (*n* = 3 and *n* = 7, respectively) to get a representative pattern of LCT*P frequencies.

In Africa, the distribution of LCT*P frequencies revealed an interesting pattern. Their correlation with longitude was not significant at the 1% level (*p* = 0.048), but we have found a positive and highly significant correlation with latitude (*r* = 0.329; *p* = 0.001). These results indicated that higher LCT*P frequencies were found in the north of the continent. Interestingly, this was where most of the African pastoral populations were located. We also calculated a linear correlation coefficient between LCT*P frequencies and pastoralism levels (PL) for 25 African populations (out of 38) for which PL data were available (Table S1) [19]. We found a very high and highly significant correlation of 0.548 (p = 0.0003), indicating that populations with a high level of pastoralism had also a high LCT*P frequency. This result was playing in favour of the *gcc* hypothesis. A Samova analysis confirmed this observation in revealing two significant boundaries which separated three groups of populations from the bulk of them: a first group including only the Fulani (with a LCT*P frequency of 100% and a PL of 80.5%), a second group composed by the Tutsi (LCT*P frequency = 74%, PL = 40.5%), various populations of Beja and the Twaregs (LCT*P frequency = 624%; average PL = 7913%), and a fourth group composed by all other populations (LCT*P frequency = 2125%, average PL = 4127%). A significant *F_CT_* of 0.44 (*p*<0.00001) was associated to this structure and was much higher than the *F_SC_* value (0.038, *p*<0.00001). This confirmed that the particular pattern of LCT*P frequencies in Africa might be explained (at least in part) by variation in the level of pastoralism.

In Europe, a positive and significant correlation has been found between genetic and geographic distances with 33 populations considered (*r* = 0.26, *p* = 0.0001, Table 1). Moreover, the correlation between LCT*P frequencies and latitude was positive and significant (*r* = 0.4, *p* = 0.001) while the correlation with longitude was not. The correlation with latitude was even higher when looking only at Indo-European populations (*n* = 26), which are considered by some authors [20] to be the descendants of the first Neolithic farmers in Europe (*r* = 0.84, *p*<0.0001). Therefore, selection on LCT*P would have occurred during the Neolithic Era, and mostly in populations from northern Europe. This fits the *cal* hypothesis whereby a vitamin-D deficiency due to insufficient sunlight in Northern Europe would have been compensated by high levels of lactose assimilation. However, we cannot exclude that the correlation found between LCT*P frequencies and latitude was due to population history rather than to natural selection, since human migrations created genetic clines along a southeast-to-northwest direction for many classical markers in Europe [21].

To tackle this last question, we first studied the correlation, in Europe, between *LCT* variation and the genetic patterns observed for two other polymorphims, RH and HLA (loci A, B and DRB1, Table 2). As mentioned above, if the evolution of LCT*P frequency was only driven by positive selection, its pattern of variation would certainly not be correlated to those of other independent genes– like RH and HLA [22] - evolving through other evolutionary forces. Our results (Table 2) revealed a high (0.42<*r*<0.53) and highly significant correlation between genetic and geographic variation for both RH [21] and HLA (all loci), as well as between both systems (although not highly significant for RH) and *LCT* (0.2<*r*<0.62). They suggested that *LCT* variation in Europe was shaped at least in part by the same evolutionary forces as for RH and HLA, e.g. those linked to population history. On the other hand, they did not allow us excluding the influence of natural selection. To further investigate this question, we used a simulation method.

**Table 2:** Comparison of LCT*P with other genetic polymorphisms (Europe).

Locus	n	r_gen,geo_	P val.	r_loc-LCT*P_	P val.
HLA-A	22	0.528	<0.0001	0.377	0.0003
HLA-B	22	0.440	0.0003	0.476	<0.0001
HLA-DRB1	14	0.460	0.003	0.620	<0.0001
RH	17	0.420	0.001	0.200	0.014

*n*: number of populations; *r*_gen,geo_: correlation coefficient between genetic and geographic distances; *r*_loc-LCT*P_: correlation coefficient between each locus and LCT*P genetic distances; *P.val*.: P-value for significance.

### Simulation

**Figure 1:**
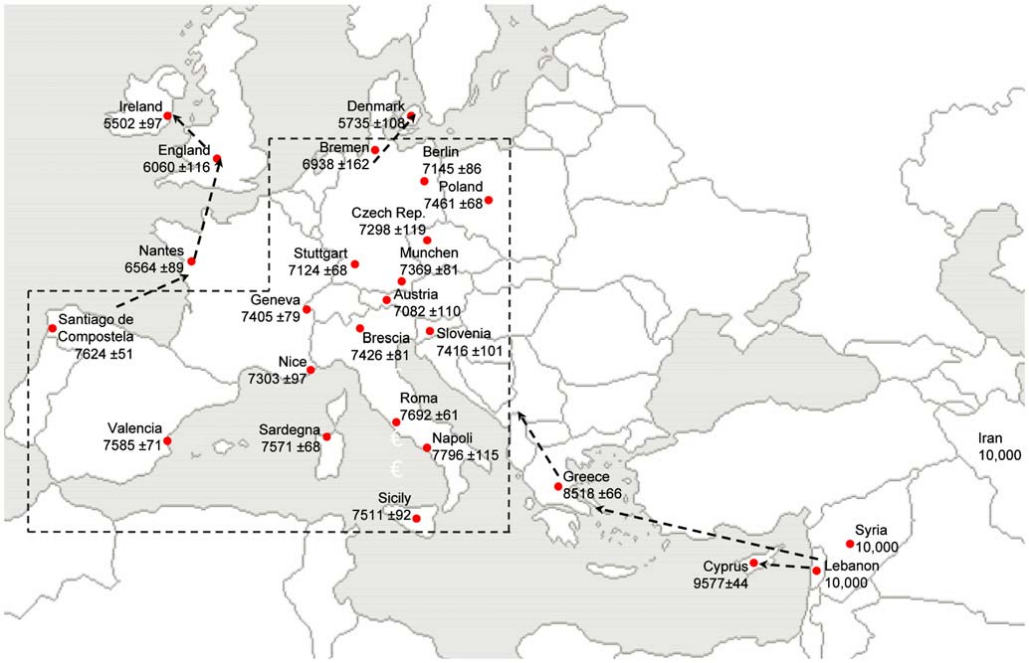
Map location of European and Near-Eastern populations used for the computer simulation. Dots locate the populations used for computer simulation (Note that the Iran sample should be located farther East, outside of the map). When more than one population from the same country were used, the name of the corresponding city is given (see Table 3). Below each population name, the approximate date of domestication and its standard error (years BP) are mentioned. When simulating the Demic Diffusion hypothesis (*DD*), dotted arrows show the simulated relations between population source and "new founded" populations (regarding initial frequency of LCT*P, see Material and Methods and Supplementary Information). To facilitate reading, the dotted area regroups populations for which we use Greece as the source.

We simulated independently for Indo-European and a few populations from the Near East the evolution of the frequency of a dominant allele associated to LP since the Neolithic transition in those populations (Figure 1). We repeated the simulation in varying the selection coefficient (*s*) for this allele, and we looked for the *s* value that explained at best the observed frequency (e.g. the value that gave the maximum likelihood) in each population. Four scenarios were simulated combining the two different demographic models described below (*CD* and *DD*) for the Neolithic transition with the two alternative hypotheses for selection at LCT*P (*gcc* and *cal*). The goal was to evaluate which of these combinations fitted at best the observed data.

**Figure 2:**
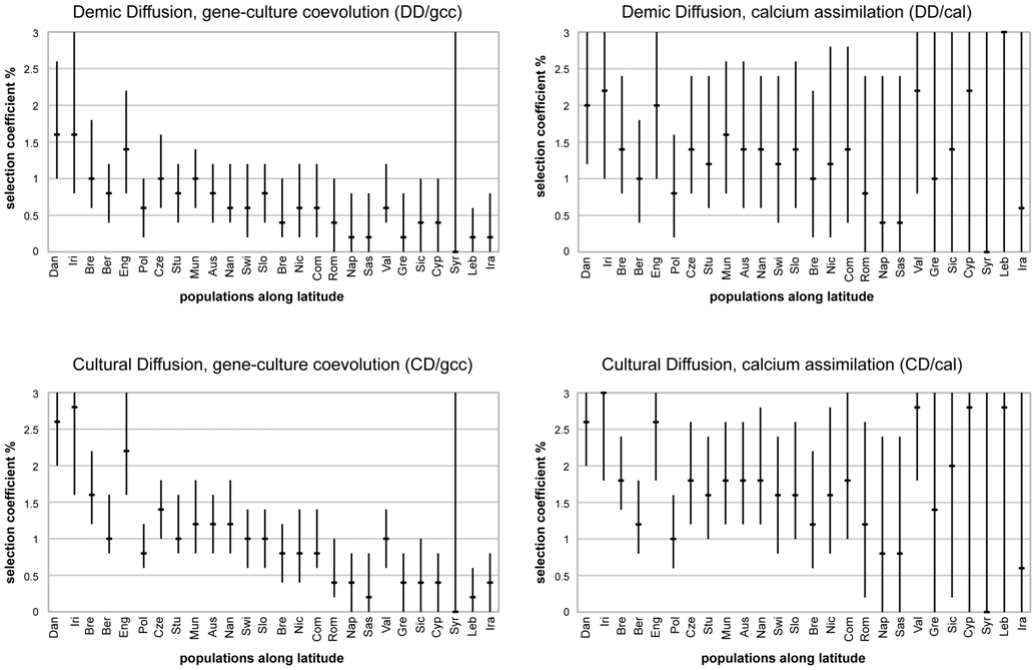
Selection coefficients required to fit the observed estimates of lactase persistence frequencies. Each graph corresponds to one of the four scenario simulated: *DD/gcc*; *DD/cal*; *CD/gcc*; *CD/cal* (see Material and Methods). Bars represent the 95% CI of the selection coefficient estimated for the population and the central point is the MLE (Maximum Likelihood Estimate). Populations are ordered from the highest (right) to the lowest (left) latitude: Danish (Dan), Irish (Iri), German from Bremen (Bre), German from Berlin (Ber), English (Eng), Polish (Pol), Czech (Cze), German from Stuttgart (Stu), German from Munchen (Mun), Austrian (Aus), French from Nantes (Nan), Swiss (Swi), Slovenian (Slo), Italian from Brescia (Bre), French from Nice (Nic), Spanish from Santiago de Compostela (Com), Italian from Roma (Rom), Italian from Napoli (Nap), Sardinian (Sas), Spanish from Valencia (Val), Greek (Gre), Sicilian (Sic), Cypriot (Cyp), Lebanese (Leb) and Iranian (Ira).

The results are presented in Figure 2 and Table S2 (supplementary information). We noticed that genetic drift alone was able to explain LCT*P frequencies in southern Europe (0 is included in the 95% CI of *s*) in all four scenarios. More precisely, the absence of selection was compatible with LCT*P frequencies in all populations located south of latitude 41°N, except Valencia (the most western, Figure 2) and Sicily (Figure 2, *CD/cal*). On the contrary, higher coefficients were required in north-western populations for all scenarios tested, except for Poland (the most eastern, Figure 2). We could also noticed that the maximum likelihood was globally higher in the north-western than in the south-eastern populations (Supplementary Information, Table S2), revealing that a model with selection was particularly well adapted to the high LCT*P frequencies of northern Europe. This result was in agreement with the correlation found between latitude and LCT*P frequencies in Europe (Table 1). Therefore, our study revealed that genetic drift alone could not explain the high LCT*P frequencies in northern and western Europeans, but that positive selection was required (under the specific model of local population demographic growth considered in this work).

Among the four tested scenarios, Demic Diffusion with calcium assimilation (*DD/cal*) had, by far, the highest probability of obtaining the observed data (99.1%), followed by *CD/cal* (0.9%). Both *gcc* scenarios (*DD/gcc* and *CD/gcc*) had an associated probability equal to 0%. The average selection coefficient for the 1,000 best simulations was 1.2% with a 95% percentile between 0.8% and 1.8%. Given the fact that all those estimations had been obtained with the *cal* selection model (which makes the assumption that selection increases with latitude), *s* thus varied across populations from 0% in the southern populations, until a maximum of 1.8% in the most Northern populations. From these observations, the interval [0.8%–1.8%] can be taken as a plausible range for LCT*P selection coefficient in Europe. Note that the estimated values of the selection coefficients were smaller when simulating the Neolithic demic diffusion (*DD)* compared to the cultural diffusion (*CD*). This may be explained by the “surfing phenomenon”, which describes how the frequency of a neutral or positively selected mutation may increase during a demographic expansion, if this mutation is carried by the pioneers [23], [24]. Computer simulations thus rejected a model of constant selection over the whole continent. In that sense, as our selection coefficients had been estimated independently, these independent estimates could have given possible intervals of the selection intensity for each population studied here. Furthermore, computer simulations showed that the high LCT*P frequencies observed in North and Western Europe may be explained by a selective pressure, possibly increasing with latitude, combined to the effect of demographic expansion during the Neolithic transition.

Alternatively to a selection increasing with latitude, Gotherstrom et al proposed that dairy farming was more intensively developed in Central European populations belonging to the early Neolithic Linearbandkeramik culture (LBK) [25]. In order to test this alternative hypothesis, we simulated two additional scenarios under the “*lbk*” selection hypothesis (*DD/lbk* and *CD/lbk*) to see if the resulting simulations better fit the data than the four previously tested scenarios. Under the *lbk* selection hypothesis, we assumed that the selection coefficient was higher in the populations belonging to the former LBK area [26] than in other populations. We tested combinations of values between 1% and 3% in the five “*lbk*” populations from our database (Munchen, Stuttgart and Berlin - all in Germany - Czech and Polish) and values between 0% and 0.8% in all other populations (see also Supplementary information for more details). 360,000 new simulations were performed under this new scheme. The Approximate Bayesian Computation approach (ABC) estimated a probability of 0% for both *DD/lbk* and *CD/lbk* scenarios compared to the four previously simulated ones. We can thus not conclude that the hypothesis of a higher selective pressure on LP in central and north Europe due to more intensive cattle exploitation is a better explanation of the current distribution of LP in Europe, compared to the *cal* and *gcc* hypotheses.

## Discussion

### Lactase persistence and population history

Analyses performed on the large dataset gathered in this work did not show any strong correlation between LCT*P genetic distances and geographic distances among populations at the worldwide scale. This suggests that the evolution of the lactase gene cannot be simply explained by a model of isolation by distance. Indeed, the significant correlation coefficients found between LCT*P frequencies, latitude and longitude are better explained by a pronounced genetic differentiation between a few geographically distant populations than by continuous patterns of frequency variation. We thus analysed our data regarding the two main selective hypotheses proposed to account for the special pattern of LCT*P distribution, *gene-culture coevolution* (*gcc*) [8], [9] and *calcium assimilation* (*cal*) [10].

The *gcc* hypothesis is favoured by the high and significant correlation found between pastoralism levels and LCT*P frequencies in 25 African populations, as well as by the Samova analysis, which reveals a significant genetic boundary between pastoral populations and the others. This result agrees with Holden and Mace's conclusion [11]. In Europe, the gradient of LCT*P frequencies could be explained either by a positive selection on this allele or by population history, or both. As comparisons between *LCT*, RH and HLA lead to quite similar results, it was possible to conclude to a significant effect of population history, but not to exclude a significant selection pressure on the lactase gene. We thus simulated the evolution of LCT*P according to various parameters (genetic drift, demographic growth, positive selection and the time since the introduction of domestication).

### Simulated scenarios and selection coefficient values

The results of our simulation show that the current frequencies of LCT*P in the North-western part of Europe are too high to be only due to genetic drift. We estimated the 95% upper limit for LCT*P selection coefficients as large as 3% in some populations (Figure 2). Our average estimates for *s* (0.8%–1.8%) are compatible with previous studies (i.e. 1.4–1.5%) [7] and also indicate that selection pressure may have been extremely different in various populations. We also confirm the fact (mentioned above) that population history played an important role in shaping the lactase persistence distribution in Europe, since the demic diffusion (*DD*) scenario associated to the *cal* selection model is the most compatible with the observed data (>99%). It is important to note that the *DD* scenarios tested here cannot be assimilated to the classical wave-of-advance model [21] because the number of steps considered for the transmission of LCT*P along the route of the Neolithic spread in Europe is low. Actually, if the number of steps was higher, and if founder effects were also considered like in the case of spatially explicit-simulations (e.g. [16]), the role of demography in increasing the frequency of LCT*P would certainly be even more pronounced. In that case, we would expect the selection coefficients to be smaller than our present estimates.

While a strong selective effect on LCT*P is suggested in Northern Europe, its cause remains to be clarified. Actually the *cal* hypothesis is possible because simulations strongly suggest a signature of selection along the latitude, but one can imagine another cause of variation for the selection coefficient, such as nutritional selection due to specific diets or economies in different regions. Indeed, while North-western European populations were the last (in continental Europe) to introduce cattle domestication, dairy farming was more intensively developed in those regions [25]. This could potentially explain the higher frequencies of LCT*P in populations from Northern Europe. However, our simulations of a higher selective pressure in the former LBK region (central and north Europe) do not support this hypothesis. An alternative hypothesis would be that a strong genetic structure for LCT*P was already present during the Mesolithic Era and lead to differential milk consumption in European populations (“The reverse cause hypothesis” [9], [27]). This hypothesis cannot formally be excluded, although the arguments favouring this scenario are yet too scarce [28].

Of course, it is important to keep in mind the limits of the model tested in this study. The choice of parameters was very difficult and we had to resort to some approximations. Using a starting value of 1%, we made the assumption that LCT*P was present but not frequent before the Neolithic transition in Europe, as suggested by the rare data available [28]. Note that this value is higher than the 1‰ used in two other studies [4], [7]. Another source of imprecision was the time elapsed since the introduction of animal domestication based on archaeological data, which often depends on indirect evidence. This problem is amplified by the confusion, in the literature, between plant and animal domestication. Also, all effective population sizes were fixed to 1,000 individuals in the first generation and increased until reaching a carrying capacity of 10,000, which is a huge simplification. The effective size of European populations since the Neolithic Era was certainly higher than 10,000; but the chosen value is conservative to detect selection, as the higher the effective population size, the weaker the genetic drift and, consequently, the smaller the probability for a rare mutation to reach a high frequency without selection. This has been confirmed with simulations using smaller population sizes (from 100 to 1,000; 500 to 2,000, results not shown). Despite our simplifications (which were the same for all populations), the main results are consistent over all sets of simulations performed. An important improvement of our study would be to take into account the spatial expansion of populations during their Neolithic settlement, as well as migration and possible admixture between hunter-gatherers and Neolithic farmers [16]. It would certainly lead to a more precise estimation of the selection coefficient. Such extensions will certainly be possible in the near future and be useful to study other potentially selected genes, like that of the Duffy blood group.

Our study highlights the difficulty to evaluate the probability of the alternative hypotheses of selection for LP in Europe because a correlation between LP frequency and latitude is expected under all of them and also because they are not mutually exclusive. Indeed, as the basic assumption is that the selective pressure on LP started at the same time than milk consumption, all three selection hypotheses tested here (*cal*, *gcc* and *lbk*) are strongly linked to the Neolithic diffusion. Both a selection coefficient increasing with latitude and a demographic spread of LCT*P would lead to identical signatures into the northern European populations compared to the southern ones. Consequently it is very difficult to disentangle the effects of demography from those of selection. One key to differentiate with more confidence the alternative selective hypotheses would be to get more samples from the Iberian Peninsula because in this particular region, LP frequency is expected to be very low under *cal* or *lbk* hypotheses and certainly higher under *gcc*. Another key would be to get more samples from other regions of the world, especially from Asia because cross-continental analyses will bring a more precise understanding of LP diffusion.

### Conclusion

If the *gcc* hypothesis is compatible with LP distribution in Africa, the *cal* hypothesis, in conjunction with demographic effects, better explained its distribution in Europe. We show that selective pressure on LP was stronger in Northern Europe than in the South. This could possibly be explained by the fact that carriers of the lactase persistence allele(s) are favoured in high-latitude regions but eventually by another reason that still needs to be determined. Moreover, we show that the European Neolithic transition played an important role in the diffusion of LP over the continent as revealed by the simulation results but also by the facts that: *i*) the correlation between *LCT* and other independent loci submitted to distinct evolutionary forces (RH and HLA) is significant; *ii*) the sample from Valencia, in the Western part of Europe, is located at low latitude (below 41°N) but has an estimated selection coefficient above 0 (see Figure 2); In this case, selection due to the latitude cannot be invoked while the frequency of LCT*P may have increased because of the Neolithic transition, Valencia being located at one extremity of this expansion process; Indeed, in case of range expansion during the Neolithic transition in Europe, the frequency of LCT*P may thus have increased gradually to a particularly large extent if this allele was maintained by selection in the early Neolithic populations, i.e. at the front of the Neolithic expansion. This hypothesis is compatible with the inferred ages for the −13,910*T mutation (between 2,188 and 20,650 years [7] and between 7,450 and 12,300 years [7],[14]). Our own results, based on statistical analyses and simulation of genetic evolution on a large dataset of LP frequencies, are thus complementary to those obtained from molecular analyses done at high resolution on this gene [6].

## Materials and Methods

### Population data

We studied 115 worldwide populations tested for LP: 38 African, 34 Asian, 33 European (among which 26 Indo-European populations), 7 American and 3 Oceanian [11], [29], [30], [31]. These data (Table S1 in Supplementary Information) were gathered from the literature. For our correlation study, we also used a large set of data on European populations tested for RH (frequencies of 8 Dd/Cc/Ee haplotypes) and HLA (allelic frequencies at loci A, B and DRB1) polymorphisms [21], [32], [33]. RH and HLA were chosen because the evolution of these systems was probably driven by different forces than *LCT*. RH may have undergone a complex selective effect due to foeto-maternal incompatibility, but its frequency patterns are highly correlated to geography at the world scale [21], suggesting a nearly-neutral mode of evolution. HLA is known to be submitted to balancing selection [34], although probably not to a similar extent at all loci [22], [33] and without blurring the signatures of population history [35], [36], [37]. A significant correlation between *LCT* and RH or HLA genetic patterns is thus expected only if these genes shared a common evolution linked, at least in part, to human population history, and not distorted too much by natural selection.

### LP (lactase persistence) frequencies

Depending on the sources consulted for this study [11], [29], [30], [31], we directly used published LCT*P frequencies or recomputed them from LP phenotypes by assuming Hardy-Weinberg equilibrium. Note that there are several methods diagnosing LP. One of the most reliable is the quantification of breath H_2_ after ingestion of lactose [30]. However, this method can be influenced by several factors like tobacco smoking or antibiotics. Moreover, malnutrition, as well as viral infections and inflammation of the intestine are known to reduce lactase expression, leading to an incorrect diagnosis [3]. Thus, one should be aware that all these factors are responsible for a certain amount of imprecision while estimating LCT*P frequencies, as it is the case in this study. However, frequency differences due to that imprecision are certainly negligible compared to the frequency differences observed between populations.

### Pastoralism levels

Pastoralism levels (PL) are used in our analysis of lactase persistence variation in Africa. These values, taken from Murdock's Ethnographic Atlas [19], roughly correspond to the percent of subsistence due to milk consumption in each population.

### Geographic coordinates and distances

The geographic coordinates of each population were used for the correlation analyses. We determined those coordinates from population localities by using the “Getty Thesaurus of Geographic Names” (http://www.getty.edu). If no locality was indicated in the literature, we used the central coordinates of the corresponding country. Those coordinates were also used to calculate geographic great-circle distances between populations using a homemade program [38].

### Genetic Distances

For a given locus, genetic distances between two populations were calculated according to where *x_i_*_,_ and *y_i_* are the frequencies of allele *i* in the populations *x* and *y*, respectively, and *k* is the total number of alleles at that locus [39].

### Samova

The Samova analysis [40] allows to determine genetic boundaries between *n* populations by using both their geographic location and their allelic frequencies at a given locus. The algorithm tries to find the distribution of populations among *k* groups defined *a priori* that maximises the *F_CT_* index (proportion of total genetic variation due to differences among groups of populations). The group structure is modified 10,000 times and the largest *F_CT_* value is retained as the best structure. The *F_SC_* value (proportion of genetic variation explained by differences between populations within each group) associated to the optimal structure, as well as *p* values for both indexes, are also given.

### Correlations

We calculated a linear correlation coefficient (*r*) between allele frequencies and either latitudes or longitudes, and we assessed its significance by using a Student's *t* test [41]. As significance may be inflated using a *t* test due to a possible non-independence between the data points (allele frequencies of different populations may share some complex pattern of covariance driven by population history), we chose a significance level of 1% (rather than 5%) to reduce type I errors. We also performed Mantel tests [42] to assess the significance of the correlation between genetic and geographic distance matrices, as well as between two genetic distance matrices obtained for two different genetic systems.

### Introduction of domestication in European populations

To provide a lower limit for the beginning of milk consumption in each European population, we used a large documented archaeological database including the earliest dates for domestication in the different regions of Europe [43]. We determined for each population a specific date for the introduction of domestication based on detailed archaeological studies performed at proximity of the population's geographic location (Table 3). We can notice from the standard errors associated to the estimated dates (Figure 1) that domestication started at a distinct time in each region. It is worth mentioning, also, that these dates correspond to the introduction of either animal or plant domestication, or both, because the distinction between the two is not always possible from the archaeological records. Moreover, we considered that animal domestication started in the Near East around 10,000 years BP (Before Present), a likely estimation for cattle [44], [45]. Finally, we used a generation time of 27 years [46] to calculate the number of generations during which LP was potentially selected since the beginning of milk consumption in Europe.

**Table 3:** Parameters for simulated populations.

N	Population (location)	Lat	Long	Lactase persistence frequency	Simulated number of generation	L values
105	Iranian	32.0	53.0	0.171	370	0.000
225	Lebanese	33.8	35.8	0.111	370	0.075
75	Syrian	35.0	38.0	0.046	370	0.126
67	Cypriot	35.0	33.0	0.152	355	0.126
100	Sicilian	37.5	14.0	0.157	278	0.230
200	Greek	39.0	22.0	0.134	315	0.293
119	Spanish (Valencia)	39.5	−0.4	0.471	281	0.314
100	Sardinian (Sassari)	40.7	8.6	0.073	280	0.364
178	Italian (Napoli)	40.8	14.3	0.082	289	0.368
839	Italian (Roma)	41.9	12.5	0.171	285	0.414
338	Spanish (Santiago de Compostela)	42.9	−8.6	0.417	282	0.456
55	French (Nice)	43.7	7.3	0.352	270	0.490
208	Italian (Brescia)	45.6	10.2	0.286	275	0.569
153	Slovenian (Ljubljana)	46.1	14.5	0.490	275	0.590
51	Swiss (Geneva)	46.2	6.2	0.423	274	0.594
102	French (Nantes)	47.2	−1.6	0.516	243	0.636
528	Austrian (Innsbruck)	47.3	11.4	0.551	262	0.640
221	German (Munchen)	48.1	11.6	0.632	273	0.674
136	German (Stuttgart)	48.4	9.1	0.515	264	0.686
200	Czech (Plzen)	49.8	13.4	0.646	270	0.745
275	Polish	52.0	20.0	0.388	276	0.837
246	German (Berlin)	52.5	13.4	0.527	265	0.858
162	English (Birmingham)	52.5	−1.9	0.776	224	0.858
441	German (Bremen)	53.1	8.8	0.714	257	0.883
50	Irish (Dublin)	53.3	−6.3	0.800	212	0.891
761	Danish (Copenhagen)	55.9	12.4	0.827	204	1.000

*n*: sample size; *Lat*: latitude; *Long*: longitude; *L values*: coefficient used to increase the strength of selection with latitude as follow *s_Lat_* = *sL* where *s_Lat_* is the simulated selection coefficient in one given population and *s* the coefficient of selection tested overall (see Supplementary Information for further details).

### Computer simulation program

We investigated a possible effect of positive selection on LCT*P evolution in Europe by estimating which values of selection coefficient were necessary to explain the observed LCT*P frequencies. To that aim, we used a homemade simulation software called SELECTOR. Our forward algorithm takes into account genetic drift, demographic growth, positive selection, the time elapsed since the possible start of milk consumption in different regions of Europe, and various initial LCT*P frequencies. The program simulates the evolution of a positively selected allele in a population of *N* diploid individuals. Each step of the simulation corresponds to a discrete generation and each genotype of the current generation is drawn by considering the allele frequency in the precedent generation. In order to reflect the advantage conferred to LP, the fitness associated to homozygotes or heterozygotes for LCT*P is set to 1 while the fitness associated to the alternative genotype is set to 1-*s*, where *s* is the selection coefficient. Further details about the algorithm are given in the Supplementary Information.

For each simulated population, the input parameters are the following:

- The *number of generations* since the introduction of plant or animal domestication in the population (Figure 1, Figure S1 and Table 3).
- The initial LCT*P frequency: this parameter was sampled out of a normal distribution with a mean of 1% for populations from the Near-East (Syrian, Lebanese, Iranian). For all other populations, this mean was either kept constant or given different values according to the scenario simulated (*CD* or *DD*, see below).
- The *size of the populations*: the demographic growth was logistically regulated within each population (see Supplementary Information). Moreover, results were also obtained for a constant population size (Figure S2 in Supplementary Information for details).
- The *selection coefficient*: for each population from Europe and the Near-East, a range of *selection coefficients* (between 0% and 3%) was tested in order to find which value(s) best explained the observed LCT*P frequencies. Depending on the selective model considered, the tested selection coefficient was either kept constant for all simulated populations (*gcc* model) or increased with *L*, a factor related to the latitude of a population (*cal* model, see Table 3 and Supplementary Information). Additionally, a third scenario was considered (*lbk* model), in which a combination of two selection coefficients were tested to represent the hypothesis that dairy farming was more intensively developed in Central European populations belonging to the early Neolithic Linearbandkeramik culture (LBK) (see discussion). To this aim, our European samples was separated in two population groups: the ones that are located on the geographic area of the former Neolithic LBK culture, where the selection coefficients vary between 1% and 3%, and all the other populations, in which the selection coefficients vary between 0% and 0.8%.
- The *number of iterations*: the program was run 10,000 times for each selection coefficient tested to obtain an empirical distribution of frequencies expected under each scenario.

Following Currat et al. [47], we performed a maximum likelihood test (independently in each population) to compare the observed LCT*P frequencies to those simulated by our program, and to evaluate which selection coefficient(s) best explained the observed data. Both the value of *s* giving the highest proportion of simulations “compatible” with the observed frequency (i.e. the maximum likelihood value of *s*) and its 95% CI were determined (See Supplementary Information for further details).

### Simulated scenarios

In order to consider possible uncertainties about the exact impact of the Neolithic transition on the European genetic pool [48], two alternative extreme demographic scenarios were simulated:

1. Cultural diffusion (*CD*): this model makes the assumption that the Neolithic transition was a cultural transmission of technologies without large movement of populations (by opposition to the Demic Diffusion model below). It implies that the know-how of the early farmers travelled over Europe from the Near East but not their genes. Consequently, all populations are simulated without any connection between them (i.e. possible migrations). In this case, our computer simulation program draws an initial frequency of LCT*P from a normal distribution with a mean equal to 1% in every population. We thus assumed that LCT*P was rare all over Europe and the Near East (average frequency of 1%) before the Neolithic transition. According to this model, simulated populations only differ among each other by the duration of selection since the introduction of farming (Table 3).
2. Demic diffusion (*DD*): this model makes the assumption that the Neolithic transition was mainly due to the spread of early farmers from the Near East to the North and Western edges of Europe. It implies that the genes of the first farmers travelled over Europe during the Neolithic transition. To simulate this spread of genes, populations are connected to each other along the likely route of the Neolithic diffusion (Figure 1 and Figure S1 in supplementary information). Except for the three Near Eastern populations (Syrian, Lebanese and Iranian), all other populations are associated to a Neolithic source population. For the Near Eastern populations, the initial LCT*P frequency is drawn from a normal distribution with a mean equal to 1%; while for the other populations the initial LCT*P frequency is taken from a source population at the generation corresponding to the beginning of plant or animal domestication. This latter varies in each target population (Table 3 and Figure S1 in Supplementary Information). The frequency of LCT*P is thus transmitted from one source population to the next along the likely route of the Neolithic spread, assuming a migration of people diffusing their new technology. The difference between each simulated population is both the duration of selection since the introduction of farming (Table 3 and Figure S1 in Supplementary Information) and the initial frequency of LCT*P. Note that we use precise dates for the beginning of the Neolithic to connect the populations together instead of using spatial coordinates as it has been done in other contexts [16]. It results in a reduced number of migration steps (a maximum of 5 between Lebanon and Ireland). Note also that there are no more migrations between the source and the target populations once the latter as been founded.

These two alternative demographic scenarios were simulated in combination with the three selection hypotheses *gcc*, *cal* and *lbk* (see below).

Initially, four alternative extreme scenarios were simulated:

1. *DD/gcc*: Demic diffusion with gene-culture coevolution hypothesis; the frequency of LCT*P is transmitted from one population to the next (Figure 1 and Figure S1) along the presumed route of Neolithic diffusion assuming a migration of people spreading their know-how. Additionally, for all simulated populations, the value of the selection coefficient is kept constant assuming that the selective pressure on lactase persistence is equal all over the continent.
2. *DD/cal*: Demic diffusion with calcium assimilation hypothesis; populations are connected to each other and the selection coefficient increases with latitude (Table 3 and Supplementary information).
3. *CD/gcc*: Cultural diffusion with gene-culture coevolution hypothesis; there is no migrations between populations and the selection coefficient is kept constant all over Europe.
4. *CD/cal*: Cultural diffusion with calcium assimilation hypothesis; in this case, there is no migration between populations and the selection coefficient increases with latitude.
  Then, two additional scenarios were performed as follow:
5. *DD/lbk*: Demic diffusion with LBK dairy hypothesis (see discussion); The frequency of LCT*P is transmitted from one population to the next (Figure 1 and Figure S1) and a combination of two different selection coefficients are used for each simulation: a higher *s* (1–3%) for the LBK group and a smaller *s* (0–0.8%) for all other populations.
6. *CD/lbk*: Cultural diffusion with dairy farming hypothesis; There is no migration between populations and a higher selection coefficient is simulated in populations belonging to the LBK culture than in other populations.

### Scenario comparison

We formally compared the 4 simulated scenarios (then 6 including *lbk*) using the Approximate Bayesian Computation approach (ABC). For each simulation (whatever scenario is considered), Euclidian distances between simulated and observed frequencies are calculated according to Beaumont et al. [49], using a weighted multiple linear regression. Allele frequencies (LCT*P) in the 26 samples are used as summary statistics, as suggested by Sousa et al [50]. From the 640,000 simulations realized (1,000,000 when comparing 6 scenarios instead of 4), the best 1,000 are retained (those with simulated LP frequencies the closest to the observed ones). The probability of obtaining the observed data under alternative scenarios is given by the proportion of the 1,000 best simulations generated by each of those scenarios. We used the software abcEst [51] to perform these comparisons.

## Supporting Information

Electronic Supplementary Information S1 (0.10 MB RTF)

Figure S1 Schematic view of the connections between populations according to the Demic Diffusion (DD) model. Arrows represent links between Neolithic source populations and target populations. Numbers represent the generations elapsed since the start of the Neolithic transition in the Near-eastern populations. For each population, the red curve represents the logistic demographic growth, and the green curve represents the evolution of LCT*P frequency. Note that these are not the simulated curves but schematic curves, as the LCT*P frequency evolves in many different ways depending on the parameters. Population names are as in Figure 1. Names in bold correspond to source populations.
(1.59 MB TIF)

Figure S2 Results for simulations run under constant population size of 10,000 individuals. Selection coefficients required to fit the observed estimates of lactase persistence frequencies, according to the four scenarios simulated: DD/gcc; DD/cal; CD/gcc; CD/cal (see Material and Methods). Bars represent the 95% CI of the selection coefficient estimated for the corresponding population and the central point is the MLE (Maximum Likelihood Estimate, see Table S2). Populations are ordered from the highest (right) to the lowest (left) latitude (see Figure 2).
(6.83 MB TIF)

Table S1 List of samples tested for lactase persistence phenotype (LP) and used in this study.
(0.58 MB RTF)

Table S2 Maximum likelihood values and associated selection coefficients, according to the simulated scenarios.
(0.25 MB RTF)
