# Supplementary material for: Impact of Selection and Demography on the Diffusion of Lactase Persistence
Source: PLoS One. 2009 Jul 24;4(7):e6369. doi: 10.1371/journal.pone.0006369 (PMC2711333; doi:10.1371/journal.pone.0006369)
Supplement: Electronic Supplementary Information S1 — (0.10 MB RTF) [file pone.0006369.s001.rtf]

Electronic Supplementary Information

Details of the simulation algorithm:
To estimate which values of selection coefficients would be necessary to explain the high lactase persistence frequencies observed in Europe, we used a homemade simulation software called SELECTOR. Our forward algorithm takes into account genetic drift, demographic growth, positive selection, as well as the time elapsed since the possible start of milk consumption in different regions of Europe and various LCT*P initial frequencies. The program simulates the evolution of a positively selected allele in a panmictic population of N diploid individuals. Each step of the simulation corresponds to a discrete generation. The genotype of each of the N individuals of the current generation is obtained separately by randomly choosing a paternal and a maternal allele among all the alleles of the previous generation. In order to reflect the advantage conferred by the lactase persistence phenotype, a random number (between 0 and 1) is drawn for each new genotype. If the random number is superior to the fitness associated to that genotype, then another genotype is drawn. The fitness associated to the dominant lactase persistence genotypes (homozygote or heterozygote for LCT*P) is 1, while the fitness associated to the third genotype (lactase non-persistent individual) is 1-s, where s is the selection coefficient. 

Complement of information regarding the parameters of this program: 
a)	The number of generations: it is maximal for populations from the Near-East (370 generations) and varies down to a minimum of 204 generations for North-Western European populations, following estimated dates for the Neolithic diffusion process (Figure 1, Table 3). A generation time of 27 years was used [1].
b)	The initial lactase persistence frequency: this parameter is sampled out of a normal distribution with a given mean. This mean is always set to 1% for the Near-Eastern populations (Syrian, Lebanese, Iranian). When simulating acculturation scenarios (i.e. Cultural Diffusion, see main article), this parameter is also set to 1% for all populations over Europe. We thus made the assumption that LCT*P was present but not frequent in small-size Mesolithic populations from Europe, as suggested by the rare data available [2]. When simulating replacement scenarios (i.e. Demic Diffusion, see main article), each population is given, as its initial frequency, the current frequency of the population from which it derives according to our migration model (Figure 1 and Figure S1). Assuming a Demic diffusion process for the Neolithic transition, allele frequency is transmitted between neighbouring populations, but the precise choice of the migration routes was made using archaeological information (see Figure 1). Actually, we considered only one block for all populations for which Neolithic dates are comprised between 8,500BP and 6,600BP (dotted area in Figure 1) were assigned, because establishing explicit routes in this case would have required too many assumptions. One particular choice - Santiago de Compostella as a Neolithic source for Nantes, which in turn is a Neolithic source for England - deserves more explanation: Neolithic influences on the British islands may have come both from the Iberia along the Atlantic coast and Northern Europe and/or from France [3]. Consequently, for our simulations, we have arbitrarily chosen one route, among all possible. For the DD scenario, we tried several different routes to northern and western peripheric populations in order to check the robustness of our results, but it did not change them. Finally, note that we do not consider founder effects in our model. 
c)	The size of the populations: the demographic growth is logistically regulated within each population as follows:  , where Nt is the density at generation t, r is the growth rate and K is the carrying capacity. In this study, N0 (the size of the population at generation 0), r and K were fixed to arbitrary but plausible values of 1,000, 0.25 and 10,000, respectively (see Discussion). Results have also been obtained for a constant population size of 10,000 individuals (Figure S2).
d)	The selection coefficients: concerning calcium assimilation (cal) simulated scenarios, a tested selection coefficient varies with L, a coefficient related to latitude. L varies between 0 and 1 such as:  where latMIN is the latitude of the population living at the lowest latitude, latMAX is the latitude of the population living at the highest latitude, and lati is the latitude of population i (Table 3). For a given population, the tested selection coefficient s is modified according to sLAT = s.L. Thus, for any value of the tested selection coefficient, s stays around 0 for populations living at lowest latitudes, and increases to its tested value for populations living at higher latitudes. As a more practical example, if the tested selection coefficient s is 3% for a simulation, s equals 0.00225 in Lebanon, 0.00879 in Greece, and increases gradually to reach its tested value of 3% in Denmark. 
Additionally to gcc and cal, a third scenario was tested, called lbk, for Linearbandkeramik, which can be considered as intermediate between the latter two. This hypothesis considers the possibility that the selection coefficient has been higher in the Linearbandkeramik culture, for which archaeological data tend to suggest a higher development of dairy farming. To simulate this lbk scenario, our population samples were separated into two groups: a Northern group where the Neolithic Linearbandkeramik culture is likely to have been of importance and another group formed by all other populations. Our lbk group is made of our samples from Munich, Berlin, Stuttgart, Poland and Czech Republic. In this scenario the lbk group is given a higher selection coefficient than the other group. More precisely speaking, the range of selection coefficients for the lbk group is taken between 1% and 3%; while for the other populations the selection coefficients is taken between smaller values: 0% to 0.8%. Thus, for one simulation under the lbk model, a combination of two selection coefficients are tested. 
e)	The number of iterations: the program was run 10,000 times for each selection coefficient tested, in order to obtain an empirical distribution of expected frequencies under the model defined by the given number of generations and selection coefficient. 

Following Currat et al. [4], we performed a maximum likelihood test to compare the observed lactase persistence frequencies to those simulated by our program, in order to determine which value(s) of the selection coefficient best explained the observed data. Each population was tested independently. Briefly, we considered as “compatible” simulations those for which the frequency of the selected allele fell into the 95% confidence interval (CI) around the observed frequency (estimated as the observed frequency plus or minus two standard errors). The simulation result is an approximation of the probability distribution of the observed data (i.e. the likelihood). We estimated the value of s for which the likelihood was maximal (i.e. the one giving the highest proportion of “compatible” simulations, the maximum likelihood estimation (MLE)). The lower and upper limits of the 95% CI of s correspond approximately to the critical values given by the likelihood distribution when 2 log-likelihood units (if only the s parameter is varying), or when 3 log-likelihood units (if both s and the initial frequency vary) are subtracted to the MLE [5].


Figure S1. Schematic view of the connections between populations according to the Demic Diffusion (DD) model. Arrows represent links between Neolithic source populations and target populations. Numbers represent the generations elapsed since the start of the Neolithic transition in the Near-eastern populations. For each population, the red curve represents the logistic demographic growth, and the green curve represents the evolution of LCT*P frequency. Note that these are not the simulated curves but schematic curves, as the LCT*P frequency evolves in many different ways depending on the parameters. Population names are as in Figure 1. Names in bold correspond to source populations.


Constant population size: 

Figure S2. Results for simulations run under constant population size of 10,000 individuals. Selection coefficients required to fit the observed estimates of lactase persistence frequencies, according to the four scenarios simulated: DD/gcc; DD/cal; CD/gcc; CD/cal (see Material and Methods). Bars represent the 95% CI of the selection coefficient estimated for the corresponding population and the central point is the MLE (Maximum Likelihood Estimate, see Table S2). Populations are ordered from the highest (right) to the lowest (left) latitude (see Figure 2).
Under the constant population size model, the results of the simulation program are nearly the same than under the demographic growth model. However, the selection coefficients required to explain the observed lactase persistence frequencies are slightly higher under constant population size than under demographic growth (Table S2). Genetic drift alone is able to explain LCT*P frequencies in Southern Europe (0 is included in the 95% CI of s), whereas non-null selection coefficients are required to explain the high LCT*P frequencies in Western and especially Northern Europe, in all scenarios but one (CD/gcc). Demic diffusion tends to decrease the selection coefficients required to fit the observed LCT*P frequencies compared to the Cultural diffusion (Table S2). 


Table S1. List of samples tested for lactase persistence phenotype (LP) and used in this study. 

Table S2. Maximum likelihood values and associated selection coefficients, according to the simulated scenarios. 


References :
1. Gage TB (1998) The comparative demography of primates: with some comments on the evolution of life history. Annual Review of Anthropology 27: 1841-1853.
2. Burger J, Kirchner M, Bramanti B, Haak W, Thomas MG (2007) Absence of the lactase-persistence-associated allele in early Neolithic Europeans. Proc Natl Acad Sci U S A 104: 3736-3741.
3. Mazurié de Keroualin K (2003) Genèse et diffusion de l'agriculture en Europe : agriculteurs, chasseurs, pasteurs. Paris: Errance. 184 p.
4. Currat M, Trabuchet G, Rees D, Perrin P, Harding RM, et al. (2002) Molecular Analysis of the beta-Globin Gene Cluster in the Niokholo Mandenka Population Reveals a Recent Origin of the betaS Senegal Mutation. Am J Hum Genet 70: 207-223.
5. Kalbfleisch JG (1985) Probability and Statistical Inference. New York: Springer Verlag.
6. Flatz G (1987) Genetics of lactose digestion in humans. Adv Hum Genet 16: 1-77.
7. Bowman JE, Murray RF (1990) Genetic variation and disorders in peoples of African origin. Baltimore, London: John Hopkins University Press.
8. Sabbagh A (2002) Etude de la diversité génétique dans les populations humaines de gènes sélectionnés [DEA in biodiversity: genetics, history and evolutionary mechanisms]. Paris: University of Paris VI, VII, IX, MNHN, INAPG.
9. Roychoudhury AK, Nei M (1988) Human Polymorphic Genes World Distribution; Press Ou, editor. New york - Oxford. 400 p.
10. Holden C, Mace R (1997) Phylogenetic analysis of the evolution of lactose digestion in adults. Hum Biol 69: 605-628.
