# Supplementary figures and images for: Impact of Selection and Demography on the Diffusion of Lactase Persistence
Source: PLoS One. 2009 Jul 24;4(7):e6369. doi: 10.1371/journal.pone.0006369 (PMC2711333; doi:10.1371/journal.pone.0006369)

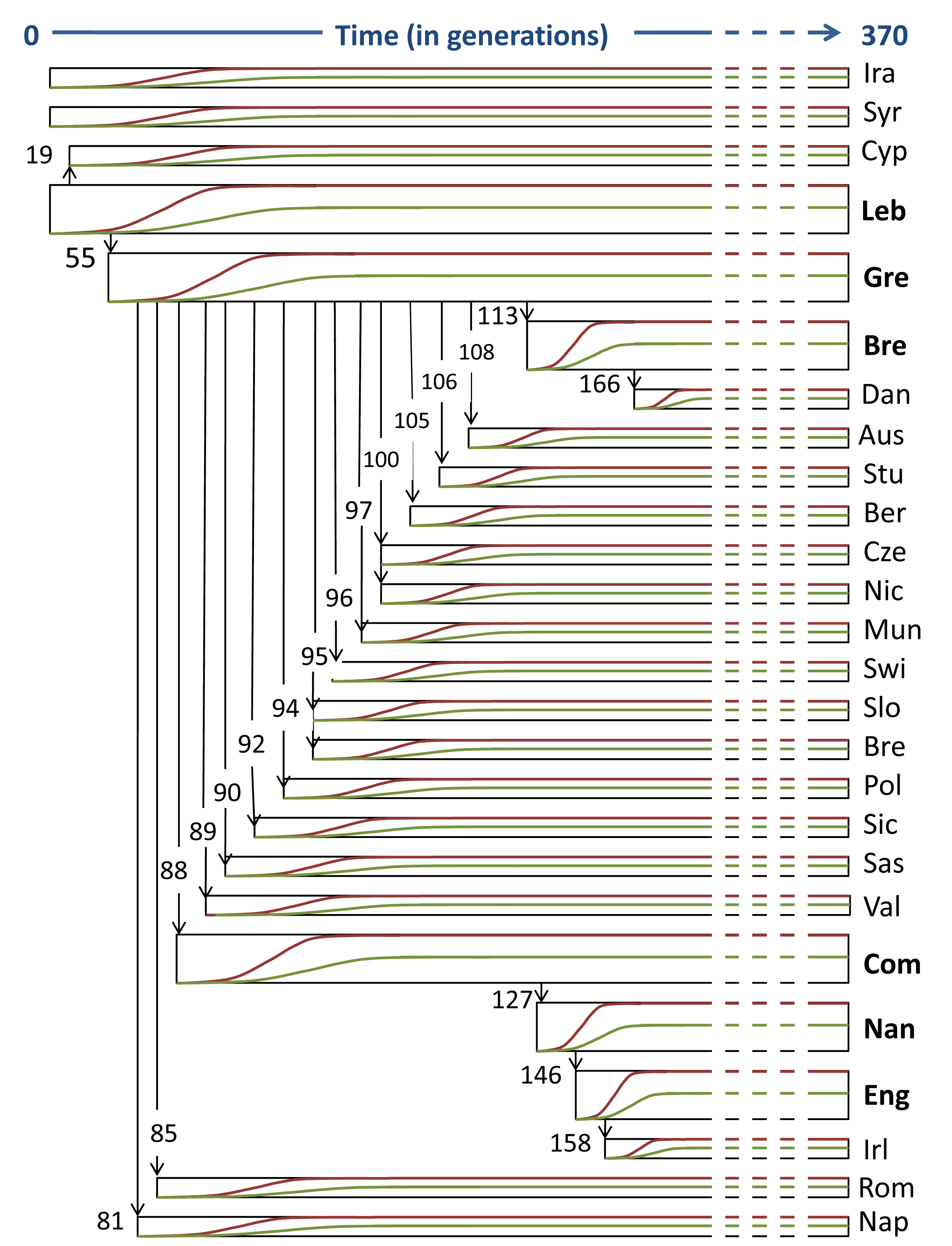

Supplement: Figure S1 — Schematic view of the connections between populations according to the Demic Diffusion (DD) model. Arrows represent links between Neolithic source populations and target populations. Numbers represent the generations elapsed since the start of the Neolithic transition in the Near-eastern populations. For each population, the red curve represents the logistic demographic growth, and the green curve represents the evolution of LCT*P frequency. Note that these are not the simulated curves but schematic curves, as the LCT*P frequency evolves in many different ways depending on the parameters. Population names are as in Figure 1. Names in bold correspond to source populations. (1.59 MB TIF) [file pone.0006369.s002.tif]

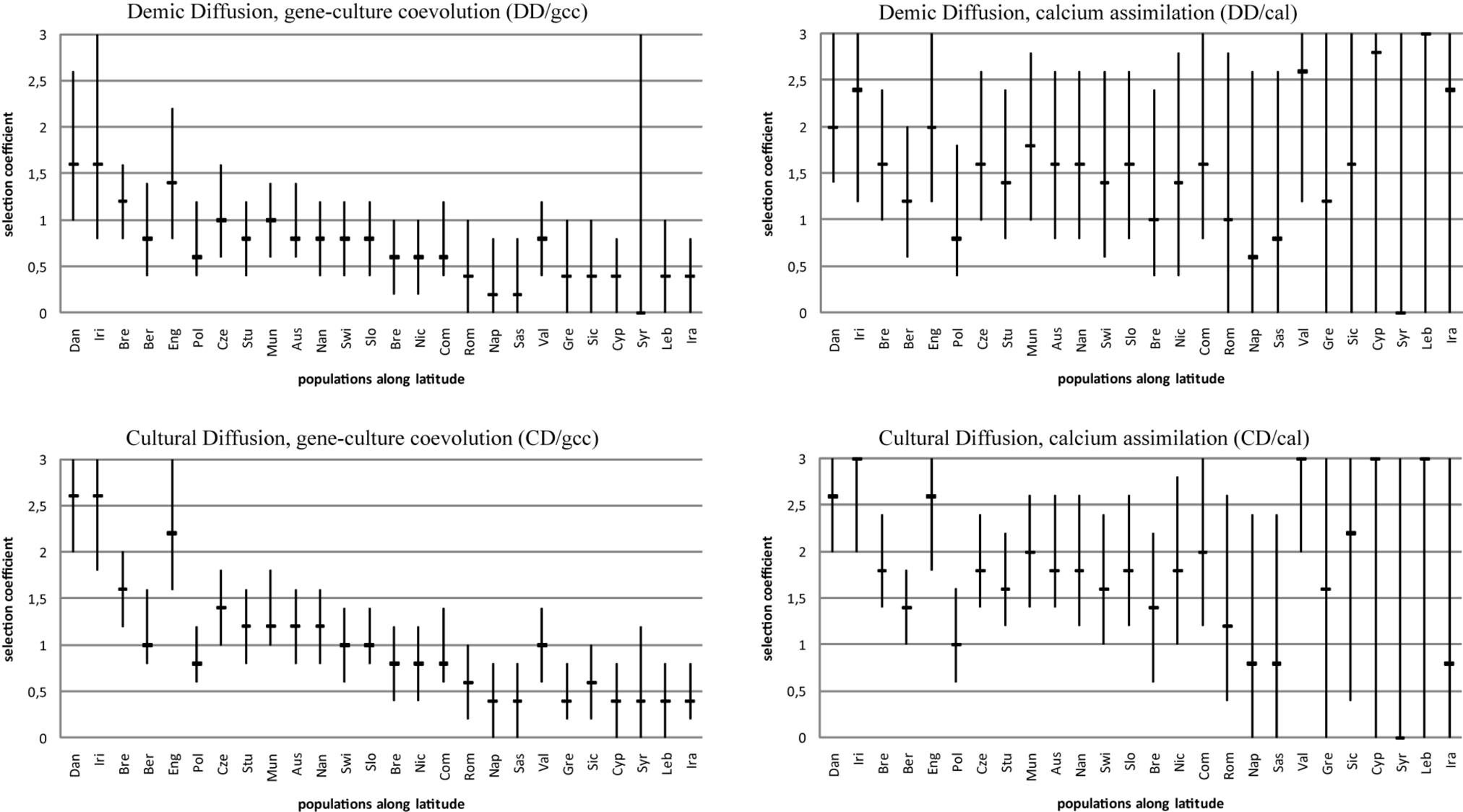

Supplement: Figure S2 — Results for simulations run under constant population size of 10,000 individuals. Selection coefficients required to fit the observed estimates of lactase persistence frequencies, according to the four scenarios simulated: DD/gcc; DD/cal; CD/gcc; CD/cal (see Material and Methods). Bars represent the 95% CI of the selection coefficient estimated for the corresponding population and the central point is the MLE (Maximum Likelihood Estimate, see Table S2). Populations are ordered from the highest (right) to the lowest (left) latitude (see Figure 2). (6.83 MB TIF) [file pone.0006369.s003.tif]
