# Supplementary material for: Impact of Selection and Demography on the Diffusion of Lactase Persistence
Source: PLoS One. 2009 Jul 24;4(7):e6369. doi: 10.1371/journal.pone.0006369 (PMC2711333; doi:10.1371/journal.pone.0006369)
Supplement: Table S1 — List of samples tested for lactase persistence phenotype (LP) and used in this study. (0.58 MB RTF) [file pone.0006369.s004.rtf]

Table S1. List of samples tested for lactase persistence phenotype (LP) and used in this study. 
Country	Population	Continent	Lat.	Long.	n	LP	P.L.6	References	
Afghanistan	Afghans Pashtoons (Kandahar)	Asia	31.6	65.7	71	0.11		Rahimi et al. 1977[6]	
Afghanistan	Afghans Tajik Hazara (Bamian)	Asia	34.8	67.8	79	0.09		Rahimi et al. 1978[6]	
Afghanistan	Afghans Pasha-I (Kaboul)	Asia	34.5	69.1	60	0.07		Rahimi et al. 1979[6]	
Australia	Native Australians	Oceania	-25	135	n.a.	0.08		Brand et al. 1983[7]	
Austria	Austrians (Innsbruck)	Europe (IE)	47.3	11.4	528	0.55		Rosenkranz, 1982[8]	
Bangladesh	Bengalis	Asia	24	90	234	0.10		Brown et al. 1979[9]	
Bolivia	Aymara (La Paz)	America	-15.5	-68	122	0.21		Balanza & Taboada 1985[9]	
Burundi	Hutu	Africa	-3.5	30	51	0.29	30.5	Cook & Kajubi 1966[10]; Cox & Elliott 1974[10]	
China	Han (North)	Asia	32.5	-114	148	0.04		Wang et al. 1984[9]	
China	Han (North)	Asia	32.5	-114	248	0.04		Wang et al. 1984[7]	
Cyprus	Greek Chypriots	Europe (IE)	35	33	67	0.15		Kattamis et al. 1973[6]	
Colombia	Chami	America	-17	-65	24	0.24		Alzate et al. 1969[9]	
Czech Republic	Czech (Plzen)	Europe (IE)	49.8	13.4	200	0.65		Madzarovova-Nohejlova, 1974, 1982 [8]	
D.R. Congo	Batusi	Africa	0	25	n.a.	1.00		Simoons 1981[7]	
D.R. Congo	Shi	Africa	0	25	n.a.	0.02		Simoons 1981[7]	
Denmark	Eskimos	Europe	72	-40	119	0.08		Gudmand-Hoyer & Jarnum 1969 [10]; Gudmand-Hoyer et al. 1973 [10]; Asp et al. 1975 [10]	
Denmark	Danish (Copenhagen)	Europe (IE)	55.9	12.4	761	0.83		Gudmand-Hoyer, 1969 [8]; Busk, 1975 [8]	
Egypt	Egyptians (Alexandria)	Africa	31.2	29.9	388	0.15		Halstead et al. 1969 [6] 
;Hussein et al. 1982[6]	
Egypt	Egyptians (Assiout)	Africa	27.2	31.1	111	0.08		Halstead et al. 1969 [6] 
;Hussein et al. 1982[6]	
Egypt	Egyptians (Assouan)	Africa	24	32.9	85	0.23		Halstead et al. 1969 [6] 
;Hussein et al. 1982[6]	
Egypt	Nubians (Nidobi)	Africa	22	34	21	0.18	93.0	Bayoumi et al. 1981 [10]	
Estonia	Estonians (Paide)	Europe	59	25.5	776	0.50		Sahi, 1994 [8]	
Fidji islands	Fijians	Oceania	-18	178	12	0.00		Masarei et al. 1972 [10]	
Finland	Finns (Jyväskylä)	Europe	62.3	25.8	449	0.58		Sahi, 1974 [8];
Jussila, 1969, 1970 [8]	
Finland	Saami (Skolt, Fisher)	Europe	62.3	25.8	176	0.23		Isokoshi et al. 1981 [10]	
France	French (Nantes)	Europe (IE)	47.2	-1.6	102	0.52		Cloarec, 1991 [8]	
France	French (Nice)	Europe (IE)	43.7	7.3	55	0.35		O'Morain, 1978 [8]	
Germany	Germans (Bremen)	Europe (IE)	53.1	8.8	441	0.71		Rotthauwe, 1972[8];
Flatz, 1982 [8]	
Germany	Germans (Berlin)	Europe (IE)	52.5	13.4	246	0.53		Rotthauwe, 1972 [10]
Flatz, 1982 [8]	
Germany	Germans (Munich)	Europe (IE)	48.1	11.6	221	0.63		Rotthauwe, 1972 [10]
Flatz, 1982 1	
Germany	Germans (Stuttgart)	Europe (IE)	47.8	9.2	136	0.51		Rotthauwe, 1972 [10]
Flatz, 1982 [8]	
Greece	Greeks	Europe (IE)	39	22	200	0.13		Ladas et al. 1982[7]	
Hungary	Hungarians (Budapest)	Europe	47.5	19.1	535	0.39		Czeizel, 1983 [8]	
India	Indians (New Delhi)	Asia	28.6	77.2	124	0.48		Tandon et al. 1981[9]	
India	Indians (Hyperabad)	Asia	17.4	78.4	72	0.37		Reddy & Pershad 1972[9]	
India	Indians (Pondicherry)	Asia	11.9	79.8	60	0.18		Tandon et al. 1981[9]	
India	Punjabi	Asia	30	76	134	0.61		Tandon et al. 1977[9]	
Indonesia	Indonesians (Java)	Asia	-5	120	53	0.05		Surjono et al. 1973[6]	
Iran	Iranians	Asia	32	53	105	0.17		Sadre et Karbasi 1979[9]	
Irland	Irish (Dublin)	Europe (IE)	53.3	-6.3	50	0.80		Fielding, 1981 [8]	
Israel 	Ashkenazy Jews	Asia	31.5	34.8	53	0.11		Gilat et al. 1970[9]	
Israel 	Sefarad Jews	Asia	31.5	34.8	68	0.18		Gilat et al. 1970, 1973[9]	
Italia	Italians (Naples)	Europe (IE)	40.8	14.3	44	0.12		Rossi, 1997 [8]	
Italia	Italians (Rome)	Europe (IE)	41.9	12.5	839	0.17		Vernia, 2001[8]	
Italia	Italians (Sassari)	Europe (IE)	40.7	8.6	100	0.07		Meloni, 2001 [8]	
Italia	Italians (Brescia)	Europe (IE)	45.6	10.2	208	0.29		Burgio, 1985 [8]	
Italia	Italians (Naples)	Europe (IE)	40.8	14.3	178	0.08		Maiuri, 1994 [8]	
Italia	Sicilians	Europe (IE)	37.5	14	100	0.16		Burgio, 1984 [8]	
Japan	Japanese	Asia	36	138	n.a.	0.05		Nose et al. 1979[7]	
Jordany	Bedouins	Asia	31	36	162	0.51		Flatz 1987[9]	
Jordany	Jordanians	Asia	31	36	n.a.	0.20		Snook et al. 1976[7]	
Kazakhstan	Kazakhs	Asia	48	68	195	0.13		Wang et al. 1984[9]	
Kenya	Batusi	Africa	1	38	n.a.	0.60		Bayless 1976[7]	
Kenya	Masaï	Africa	1	38	n.a.	0.21	93.0	Jackson & Latham 1979[7]	
Lebanon	Libanese	Asia	33.8	35.8	225	0.11		Loiselet & Jarjouhi 1974 [10];
Nasrallah 1979 2	
Mexico	Pima (Mexico)	America	29	-108	62	0.03		Johnson et al. 1977 [10];
Johnson et al. 1978 [10]	
Mongolia	Mongols	Asia	41	112	198	0.06		Wang et al. 1984[9]	
Namibia	San (Kung & Hua)	Africa	-21	20	65	0.03	0.0	Jenkins et al. 1974 [10]; Nurse & Jenkins 1974 [10]	
Niger	Touaregs	Africa	16	8	119	0.64		Flatz et al. 1986[9]	
Nigeria	Fulani (sedentary)	Africa	10	8	24	0.16	80.5	Kretchmer et al. 1971 [10]	
Nigeria	Hausa	Africa	10	8	17	0.13	30.5	Kretchmer et al. 1971 [10]	
Nigeria	Ibo	Africa	10	8	n.a.	0.00	10.5	Johnson et al. 1981[7]	
Norway	Norwegians (Oslo)	Europe (IE)	59.9	10.8	55	0.78		Sahi, 1994 [8]	
Ouganda	Ganda	Africa	2	33	27	0.02	10.5	Cook & Kajubi 1966 [10]; Cook & Dahlquist 1968 [10]	
Ouzbekistan	Ouzbek	Asia	41	64	n.a.	0.00		Simoons 1981[7]	
Pakistan	Pathans Pushtu	Asia	33.5	70.5	86	0.19		Rab & Baseer 1976 [10]; Rahimi et al. 1976 [10]	
Pakistan	Sindhi	Asia	25.5	69	45	0.35		Rab & Baseer 1976 [10]; Ahmad & Flatz 1984 [10]	
Papua New Guinea	Papouans (west Sepik)	Asia	-4	141.5	35	0.12		Arnhold et al. 1981[9]	
Poland	Polish	Europe (IE)	52	20	275	0.39		Socha et al. 1984[9]	
Russia	Russians (Saint Petersburg)	Europe (IE)	59.9	30.3	248	0.61		Valenkevich, 1977 [8]	
Rwanda	Twi Pygmees	Africa	-2	30	n.a.	0.12	40.5	Simoons 1981[7]	
Rwanda 	Batusi	Africa	-2	30	n.a.	0.72		Arnold et al. 1980[7]	
Rwanda 	Tutsi	Africa	-2	30	59	0.74		Cook & Dahlquist 1968 [10] ; Cook & Kajubi 1966 [10] ; Cox & Elliott 1974 [10]; Elliott et al. 1973 [10]	
Saudi Arabia	Arabs	Asia	25	45	40	0.43		Cook & Al-Torki 1975[9]	
Saudi Arabia	Bedouins	Asia	25	45		0.63		Simoons 1981[7]	
Senegal	Fulani	Africa	14	-14	29	1.00	80.5	Arnold et al. 1980[6]	
Slovenia	Slovenians (Ljubljana)	Europe (IE)	46.1	14.5	153	0.49		Sahi, 1994 [8]	
Somalia	Somalians	Africa	6	48	244	0.13		Flatz & Dragonat[6]	
Sudan	Baggara (Habbaniya)	Africa	15	30	19	0.27	50.5	Bayoumi et al. 1981 [10]	
Sudan	Baggara (Messiria)	Africa	15	30	20	0.23	70.5	Bayoumi et al. 1981 [10]	
Sudan	Beja (Amarar)	Africa	15	30	82	0.64	93.0	Bayoumi et al. 1982 [10]	
Sudan	Beja (Beni Amir)	Africa	15	30	40	0.64	80.5	Bayoumi et al. 1982 [10]	
Sudan	Beja (Bisharin)	Africa	15	30	22	0.63	80.5	Bayoumi et al. 1982 [10]	
Sudan	Beja (Haddendoa)	Africa	15	30	137	0.55	60.6	Bayoumi et al. 1982[10]	
Sudan	Dinka	Africa	15	30	213	0.13	50.5	Bayoumi et al. 1982 [10]; Eliott et al. 1973 [10]	
Sudan	Kabbabish (north-west)	Africa	14	29.8	51	0.51		Bayoumi et al. 1981[6]	
Sudan	Nuer	Africa	15	30	23	0.12		Bayoumi et al. 1982 [10]	
Sudan	Shilluk	Africa	15	30	8	0.21	20.5	Bayoumi et al. 1982 [10]	
South Africa	Sotho	Africa	-29	29	23	0.19	30.5	Segal et al. 1983 [10]	
South Africa	Tswana	Africa	-28	24	24	0.09	40.5	Segal et al. 1983 [10]	
South Africa	Zulu (Kwazulu-Natal)	Africa	-28.7	30.7	n.a.	0.06	40.5	O'Keefe & Adam 1983[7]	
South Africa	Herero	Africa	-22	19	37	0.02	60.5	Currie et al. 1978 [10]	
South Africa	Nama (Khoi)	Africa	-30	26	39	0.33	50.5	Nurse & Jenkins 1977[9]	
South Africa	San/Bushmen Tsumkwe !Kung	Africa	-30	26	40	0.01	2.5	Nurse & Jenkins 1977[9]	
Spain	Spanish (Valencia)	Europe (IE)	39.5	-0.4	119	0.47		Guix Garcia, 1974 [8]	
Spain	Spanish (Compostella)	Europe (IE)	42.9	-8.6	338	0.42		Leis, 1997 [8]	
Sri lanka	Sri lankans	Asia	7	81	200	0.15		Senewiratne et al. 1977[9]	
Sri lanka	Tamouls	Asia	7	81	n.a.	0.10		Simoons 1981[7]	
Swaziland	Swazi	Africa	-26.5	31.5	12	0.13	20.5	Segal et al. 1983 2	
Sweden	Swedish (Lund)	Europe (IE)	57	13.2	400	0.78		Berg, 1967 [8]	
Switzerland	Swiss (Geneva)	Europe (IE)	46.2	6.2	51	0.42		Jodry, 1987 [8]	
Syria	Syrians	Asia	35	38	75	0.05		El-Schallah et al. 1973[6] ; Rotthauwe et al. 1971 ; Flatz 1987[9]	
Taïwan	Chinese	Asia	23.5	121	71	0.00		Sung & Shih 1972[9]	
Thailand	Thai (Bangkok)	Asia	13.7	100.5	279	0.02		Flatz 1987[9]	
Thailand	Thai (Lanpang)	Asia	18	99	149	0.00		Flatz 1987[9]	
Tunisia	Tunisians	Africa	34	9	43	0.09		Filiali et al. 1987 [10]	
Turkey	Turks (Ankara)	Asia	39.9	32.8	470	0.16		Flatz, 1986 [8]	
U.K.	English (Birmingham)	Europe (IE)	52.5	-1.9	162	0.78		Thomas, 1990 [8]	
U.S.A.	Apaches (Arizona)	America	34	-112	22	0.00		Johnson et al. 1978 [10]	
U.S.A.	Chippewa (Michigan)	America	44	-85	33	0.02		Newcomer et al. 1977 [10]	
U.S.A.	Hopi (Arizona)	America	34	-112	21	0.00		Johnson et al. 1978[10]	
U.S.A.	Papago (Arizona)	America	34	-112	14	0.04		Johnson et al. 1978[10]	
Vietnam	Vietnamese (U.S.A.)	Asia	16	106	31	0.00		Anh et al. 1977[7]	
Yemen	Arabs	Asia	15.5	47.5	n.a.	0.50		Simoons 1981[7]	
n.a.	Saami	Europe	68	25	345	0.43		Isokoshi et al. 1981[10]	
Lat: latitude; Long: longitude; n: sample size when known; LP: frequency of lactase persistence phenotype in the sample; P.L.: Pastoralism level when known; IE: Indo-European language; n.a.: not available.
	
