# Supplementary material for: Impact of Selection and Demography on the Diffusion of Lactase Persistence
Source: PLoS One. 2009 Jul 24;4(7):e6369. doi: 10.1371/journal.pone.0006369 (PMC2711333; doi:10.1371/journal.pone.0006369)
Supplement: Table S2 — Maximum likelihood values and associated selection coefficients, according to the simulated scenarios. (0.25 MB RTF) [file pone.0006369.s005.rtf]

Table S2. Maximum likelihood values and associated selection coefficients, according to the simulated scenarios. 
	Logistic growth (1,000 - 10,000 individuals)	Constant size (10,000 individuals)	
	DD	CD	DD	CD	
	gcc	cal	gcc	cal	gcc	cal	gcc	cal	
Pop	MLE	s coef	MLE	s coef	MLE	s coef	MLE	s coef	MLE	s coef	MLE	s coef	MLE	s coef	MLE	s coef	

Dan	-1.39	1.6	-1.90	2	-1.00	2.6	-0.98	2.6	-1.00	1.6	-1.33	2	-0.70	2.6	-0.69	2.6	

Iri	-0.48	1.6	-1.07	2.2	-0.22	2.8	-0.23	3	-0.12	1.6	-0.47	2.4	-0.02	2.6	-0.01	3	

Bre	-1.61	1	-2.02	1.4	-1.20	1.6	-1.22	1.8	-1.11	1.2	-1.45	1.6	-0.85	1.6	-0.84	1.8	

Ber	-1.88	0.8	-2.59	1	-1.52	1	-1.48	1.2	-1.85	0.8	-1.75	1.2	-1.25	1	-1.18	1.4	

Eng	-0.94	1.4	-1.49	2	-0.56	2.2	-0.55	2.6	-0.80	1.4	-0.84	2	-0.25	2.2	-0.25	2.6	

Pol	-2.21	0.6	-2.61	0.8	-1.82	0.8	-1.86	1	-2.05	0.6	-2.05	0.8	-1.50	0.8	-1.51	1	

Cze	-1.40	1	-1.82	1.4	-1.12	1.4	-1.11	1.8	-1.40	1	-1.25	1.6	-0.76	1.4	-0.70	1.8	

Stu	-1.61	0.8	-2.00	1.2	-1.23	1	-1.24	1.6	-1.08	0.8	-1.46	1.4	-0.92	1.2	-0.87	1.6	

Mun	-1.54	1	-1.88	1.6	-1.21	1.2	-1.17	1.8	-0.99	1	-1.37	1.8	-0.91	1.2	-0.84	2	

Aus	-2.14	0.8	-2.55	1.4	-1.84	1.2	-1.79	1.8	-2.05	0.8	-2.01	1.6	-1.42	1.2	-1.42	1.8	

Nan	-1.56	0.6	-1.93	1.4	-1.08	1.2	-1.06	1.8	-0.95	0.8	-1.31	1.6	-0.69	1.2	-0.72	1.8	

Swi	-1.37	0.6	-1.68	1.2	-1.07	1	-1.03	1.6	-0.95	0.8	-1.14	1.4	-0.66	1	-0.66	1.6	

Slo	-1.82	0.8	-2.06	1.4	-1.38	1	-1.31	1.6	-1.24	0.8	-1.52	1.6	-1.00	1	-1.02	1.8	

Bre	-2.31	0.4	-2.48	1	-1.92	0.8	-1.84	1.2	-1.75	0.6	-1.98	1	-1.52	0.8	-1.52	1.4	

Nic	-1.54	0.6	-1.83	1.2	-1.13	0.8	-1.12	1.6	-0.98	0.6	-1.27	1.4	-0.82	0.8	-0.78	1.8	

Com	-2.28	0.6	-2.57	1.4	-1.88	0.8	-1.90	1.8	-1.99	0.6	-2.03	1.6	-1.61	0.8	-1.53	2	

Rom	-3.13	0.4	-3.30	0.8	-2.64	0.4	-2.60	1.2	-2.57	0.4	-2.74	1	-2.32	0.6	-2.32	1.2	

Nap	-2.32	0.2	-2.40	0.4	-1.92	0.4	-1.80	0.8	-1.82	0.2	-1.90	0.6	-1.54	0.4	-1.54	0.8	

Sas	-2.04	0.2	-2.11	0.4	-1.50	0.2	-1.47	0.8	-1.48	0.2	-1.58	0.8	-1.25	0.4	-1.21	0.8	

Val	-1.74	0.6	-1.89	2.2	-1.36	1	-1.29	2.8	-1.16	0.8	-1.30	2.6	-0.93	1	-0.92	3	

Gre	-2.35	0.2	-2.33	1	-1.97	0.4	-1.97	1.4	-1.85	0.4	-1.98	1.2	-1.66	0.4	-1.70	1.6	

Sic	-2.09	0.4	-2.08	1.4	-1.58	0.4	-1.53	2	-1.46	0.4	-1.57	1.6	-1.27	0.6	-1.21	2.2	

Cyp	-1.80	0.4	-1.70	2.2	-1.54	0.4	-1.52	2.8	-1.29	0.4	-1.27	2.8	-1.23	0.4	-1.24	3	

Syr	-0.03	0	-0.03	0	-0.03	0	-0.03	0	-4.39	0	-0.04	0	-0.53	0.4	-0.03	0	

Leb	-2.21	0.2	-2.20	3	-2.23	0.2	-2.18	2.8	-3.20	0.4	-1.97	3	-1.95	0.4	-1.99	3	

Ira	-1.79	0.2	-3.64	0.6	-1.78	0.4	-3.61	0.6	-2.14	0.4	-3.71	2.4	-1.46	0.4	-3.70	0.8	
Maximum likelihood values (MLE) and associated selection coefficients (s coef) are presented, whether the population growth was logistically regulated (logistic growth) or the population size was constant (constant size) during all the generations simulated. Results correspond to the four scenarios simulated: Demic Diffusion and gene-culture coevolution (DD/gcc) or calcium assimilation (DD/cal); and Cultural Diffusion and gene-culture coevolution (CD/gcc) or calcium assimilation (CD/cal) (see Material and Methods). Populations (Pop) are ordered from the highest latitude (top) to the lowest (bottom). Population names are annotated as in Figures 2 and S1.
